# The Top 100 Cited Papers in the Field of Iron Deficiency in Humans: A Bibliometric Study

**DOI:** 10.1155/2021/5573790

**Published:** 2021-06-12

**Authors:** John L. Frater

**Affiliations:** Department of Pathology and Immunology, Washington University, St. Louis, MO, USA

## Abstract

Worldwide, iron deficiency is a common form of micronutrient deficiency with a high individual and societal cost. There are considerable knowledge and practice gaps in the diagnosis and treatment of iron deficiency. Bibliometric analysis examines the published body of knowledge of a subject in an objective fashion. The Web of Science Core Collection was searched to retrieve the 100 most cited papers on the topic of iron deficiency, and the key metrics of each paper were extracted. A keyword study was performed using VOSviewer 1.6.10 software, which provided a visual mapping of the network of keyword cooccurrences. The papers were published between 1964 and 2017 and were cited an average of 636 times. They were contributed by authors from 119 different countries/regions, with the largest contributing country being the United States. 29 institutions contributed at least 6 publications each, and 4 researchers authored or coauthored at least 5 papers. Keyword analysis suggests that the most cited topics could be grouped into 4 categories: (1) epidemiologic research of the global burden of iron deficiency, (2) clinical aspects of iron deficiency anemia, (3) iron metabolism, and (4) the impact of iron deficiency on children. Identification of the most impactful studies in the field of iron deficiency may be helpful to practitioners interested in improving their knowledge base. Compared to bibliometric studies performed on other topics, the medical literature of iron deficiency is mature, as evidenced by the high citation rate of the top 100 papers. Despite the high worldwide prevalence of iron deficiency, the top cited papers are dominated by a relatively small number of countries and institutions. Interestingly, however, the most cited authors in this study do not overlap with the most cited institutions.

## 1. Background

Iron deficiency is a common micronutrient deficiency, with an estimated prevalence of approximately one-third of the world population, and is thus one of the most commonly encountered abnormalities in general medical practice [1]. Although its most common clinical manifestation is a microcytic, hypochromic anemia, iron deficiency can affect a diverse range of systems including the central nervous system and reticuloendothelial system and has clinical consequences ranging from impaired immunity, cognitive deficits, decreased exercise capacity, and decreased quality of life as measured by disability-adjusted life years. Apart from its influence on individual health, iron deficiency has a profound economic impact due to impaired work capacity [2].

The field of iron deficiency is a rich one: a search of the Web of Science Core Collection (WoSCC, All Fields, search term “iron deficiency”) conducted on 28 December 2020 yielded 23,694 results. Despite the fact that iron deficiency is commonly encountered in the clinical setting, studies have shown considerable practice and knowledge gaps [3]. Such issues could be addressed using a bibliometric methodology to assess landmark studies in the history of iron deficiency research. A bibliometric study examines the published scientific body of knowledge of a given subject in an objective fashion. The purpose of this approach is to summarize the current state of the subject, examine the development of a research field, and provide guidance for future research. Although it was originally conceived as a research tool, bibliometric analysis may be of interest to any reader interested in the scope and interrelatedness of a given area of knowledge.

The WoSCC has features that make it particularly useful as a tool for bibliometric analyses. To date, there has been no comprehensive review of studies of iron deficiency using bibliometric methodology. The purpose of this study was to examine the 100 most cited papers on iron deficiency using data from the WoSCC. The data provide a comprehensive view of the current research status of iron deficiency by utilizing the year of publication, citation number, authors, country/region, organizations, journals, and other important features of these studies. The keywords from these papers were mapped using VOSviewer (Leiden University) software to demonstrate the main areas of interest in this field and their interrelations.

## 2. Methods

This study was performed using a standard bibliometric methodology, similar to that described by Lu et al. [4], and was performed using Preferred Reporting Items for Systematic Reviews and Meta-analysis (PRISMA) standards (see Supplementary Data File S1, which contains the PRISMA checklist).

### 2.1. Data Sources

The 100 most cited iron deficiency-related papers were retrieved from the WoSCC database, with no restriction on the date of publication. The search strategy used the search term “iron deficiency” in all fields with only publications indexed as “ARTICLE” or “REVIEW” being included. All hits were ranked by the number of citations.

### 2.2. Data Extraction

The author assessed the full text of each identified paper to ensure that the study referred to iron deficiency in humans. The following information was obtained for each study: title, publication year, publication type, number of citations, authors, countries/region, institutions, journals, and their impact factors (IFs). For studies originating from more than one country and/or institution, the country and institution listed in the Web of Science entry were used.

### 2.3. Statistical Analysis

Microsoft Excel 2016 software (Redmond, Washington, USA) was used for descriptive statistical analyses of year of publication, publication type, number of citations, author names, countries/regions, organizations, and journals and their IFs (as listed in the 2020 Journal Citation Reports of Clarivate Analytics) [4]. VOSviewer 1.6.10 software (Leiden University) was used to map the network of keyword cooccurrence (the unit of analysis was set to “All keywords”). In the map, each circle represents a term, the size of which indicates how often it appeared. If keywords appeared together in any of the 100 papers, a line connected the two circles; the thicker the line, the greater the number of times they appeared together. The greater the frequency of the two terms appearing together, the closer the two circles would be [5]. The resolution of the term map was set for least 7 times in the 100 papers.

## 3. Results

The 100 most cited papers in the field of iron deficiency identified in the WoSCC consisted of 67 original articles and 33 review articles. The total number of citations per paper ranged from 308 to 3877, with the mean number of citations being 636 (median = 472 citations). 46% of articles had >500 citations, and 9% had >1000 citations. Supplementary Data File S2 summarizes the data for the top 100 cited papers, organized according to number of citations and including first author name, journal, year, volume, issue, page numbers, and number of citations.

### 3.1. Year of Publication

**Figure 1:**
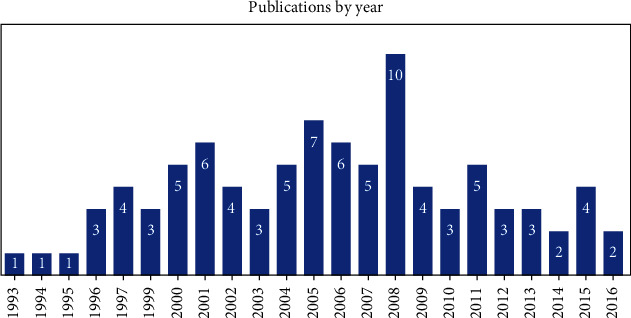
Temporal distribution of papers in this study. Two years (1964 and 2017) are off scale and are not shown.

The papers in the present study were published between 1964 and 2017. The year with the largest number of published studies was 2008.  Figure 1 presents the temporal distribution of the top 100 cited papers.

### 3.2. Distribution of Countries/Regions

The papers were contributed by authors from 119 countries/regions. Authors from 25 countries/regions contributed to at least 6 publications each (Table 1); the countries with the largest numbers of publications were the United States (71 publications), England (25 publications), Switzerland (15 publications), France (13 publications), and Italy (12 publications).

### 3.3. Distribution of Institutions

Of the institutions identified in the paper, 18 contributed at least 7 publications each (Table 2). The top 4 organizations were the University of California System, Harvard University, the University of London, and the University of Michigan.

### 3.4. Distribution of Authors

631 individuals authored or coauthored at least 2 times, and 4 researchers authored or coauthored at least 5 papers in the list. The authors appearing in 5 or more publications are B Lozoff (University of Michigan, Ann Arbor, Michigan, USA, 6 publications), R Lozano (Instituto Nacional de Salud Publica, Cuernavaca, Morelos, Mexico, 5 publications), CJL Murray (University of Washington, Seattle, Washington, USA, 5 publications), and M Naghavi (University of Washington, Seattle, Washington, USA, 5 publications).

### 3.5. Distribution of Journals

Overall, the papers in this study were published in 47 academic journals.  Table 3 lists the journals (*n* = 15) where the largest number of studies was published. The IF for the journals that published ≥2 papers ranged from 60.392 (*Lancet*) to 4.281 (*Journal of Nutrition*). These journals are published in the USA (11/15, 73%) and the United Kingdom (4/15, 27%). The largest number of papers was published by *Lancet* (17 papers), *American Journal of Clinical Nutrition* (6 papers), and *Blood* (6 papers).

### 3.6. Cooccurrence of Keywords

**Figure 2:**
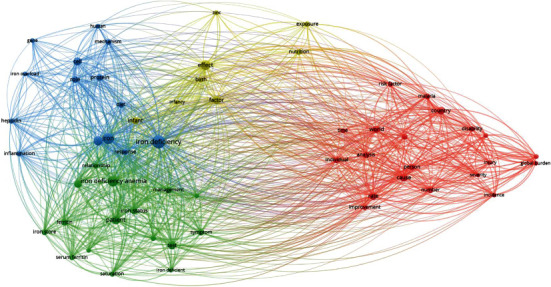
Mapping of 58 keywords occurring >7 times in papers in this study.

Overall, 58 terms appeared 7 times or more in the titles or abstracts of the top 100 papers (Figure 2). For example, “iron deficiency” appeared 48 times, “iron” appeared 33 times, “iron deficiency anemia” appeared 31 times, “effect” appeared 28 times, and “anemia” appeared 24 times. Based on this, the terms or phrases associated with iron deficiency are divided into 4 clusters, represented by 4 colors (red, green, blue, and yellow). From the results of cooccurrences, current iron deficiency research mainly focused on 4 major areas. These are (1) epidemiologic research investigating the global burden of iron deficiency (red), (2) research into the clinical aspects of iron deficiency anemia (green), (3) iron metabolism (blue), and (4) the impact of iron deficiency on children (yellow). These 4 topics may be regarded as the current research hotspots in the field of iron deficiency. The terms that appeared at least ten times are listed in Table 4.

## 4. Discussion

### 4.1. The Bibliometric Approach Applied to Iron Deficiency

This bibliometric study focuses on the 100 most cited papers related to iron deficiency research, which were published between 1964 and 2017. The purpose of this study was to evaluate the research status and trends in the field of iron deficiency research. The counties with the highest degree of representation in this study were the USA, England, Switzerland, France, and Italy with more than 10 publications each, whereas further 13 countries published at least 7 studies each between 1964 and 2017. The dominance of the USA in this research may be due to funding of basic and clinical research by groups such as the Department of Veterans Affairs and the National Institutes of Health. The University of California System and Harvard University contributed the most papers to this list, likely due to the broad interest of researchers at these multicampus academic systems in basic and clinical science regarding iron deficiency. Interestingly, the top-cited organizations did not overlap with the top-cited authors.

In addition to the high mean citation number, 9 papers were cited over 1000 times, and the lowest-cited paper was cited 308 times. The high citation impact of the top 100 papers suggests that iron deficiency is a mature research field. Using a similar methodology to the current study, the top 100 papers in drug therapy for ventilator-associated pneumonia and coronary artery disease had similar high citation numbers [6, 7], whereas an emerging field such as network pharmacology had a much lower number of papers with high citation rate and a larger delta between the highest and lowest cited papers [4]. The top 100 cited papers were published in 47 journals, among which 3 journals (*Lancet*, *American Journal of Clinical Nutrition*, and *Blood*) published over one-quarter of the papers included in this list. Most of the journals in which the top 100 cited papers were published are published in the USA and England and are generally within the fields of general and internal medicine and nutrition (see S2).

According to the results of keyword cooccurrence, the 100 most cited papers covered the main topics of iron deficiency research, including its applications and common research methods. The current research hotspots are (1) epidemiologic research investigating the global burden of iron deficiency, (2) research into the clinical aspects of iron deficiency anemia, (3) iron metabolism, and (4) the impact of iron deficiency on children. This is supported by the opinions of the authors of recent reviews in the field [8–11].

### 4.2. Epidemiology of Iron Deficiency

Iron deficiency was studied as a part of the Global Burden of Disease Study, a large-scale observational epidemiologic survey looking at 289 diseases and injuries and conducted from 1990 to the present. Four of the top 5 cited papers in the current study were published as part of this study [12–15]. Populations at increased risk of iron deficiency include persons in developing countries, individuals from lower socioeconomic classes in developed countries, and women [16–20]. Worldwide, approximately 2 billion people are anemic, of whom ~50% are iron deficient [21].

The World Health Organization estimates that the prevalence of anemia in the developing world is 39% for children < 5 years old, 48% in children between 5 and 14 years old, and 52% in pregnant women, of which approximately 50% is due to iron deficiency [22]. However, Modell and Darlison addressed a significant problem in assessing populations for estimated prevalence of iron deficiency anemia in developing nations. Since populations in these parts of the world may have an increased prevalence of thalassemia, it is important to use hemoglobin ranges specific for these populations. Using published ranges, which are predominantly drawn from patients of Northern European origin, may lead to an overestimate of iron deficiency [23].

Older populations also demonstrate an elevated risk of anemia; a study from the USA by Guralnik et al. estimates that approximately 10% of individuals over age 65 are anemic, of which half have anemia due to iron deficiency [24].

### 4.3. Clinical Features of Iron Deficiency

Iron deficiency may be caused by increased iron need, low iron intake, decreased iron absorption, or chronic blood loss [9]. In addition, chronic inflammatory conditions may predispose to the development of iron deficiency [9].

Several papers in the current study have evaluated the etiology of iron deficiency. Decreased iron absorption may be seen in patients with *Helicobacter pylori* gastritis, chronic use of nonsteroidal anti-inflammatory drugs, and celiac disease due to consequent mucosal damage [25–31]. Iron deficiency is also a common complication of bariatric surgery [32].

Among the chronic inflammatory conditions placing individuals at risk of iron deficiency, one of the best studied has been heart failure. The 2017 clinical practice guidelines for the management of chronic heart failure state that treatment of iron-depleted patients with intravenous iron resulted in quality of life improvement and better exercise capacity [33]. In the developing world, chronic inflammation associated with parasitic infection is a common cause of iron deficiency anemia. For example, hookworm infection is responsible for up to 73% of severe iron deficiency anemia in Africa [34].

Anemia is also a common complication of chronic kidney disease due to a variety of mechanisms including decreased dietary absorption due to excess hepcidin or iron deficiency due to reticuloendothelial system iron blockade [35, 36]. In response to iron deficiency, the body's absorption of lead, cadmium, and aluminum increases. The widespread consequences of increased levels of these minerals include adverse effects to the central nervous system, kidney, and skeletal system [37–39].

Iron deficiency also has widespread adverse effects on immunity, including impairment of B-cell and T-cell function and decreased phagocytosis and macrophage killing power [40]. Iron deficiency is associated with increased risk of malaria and increased morbidity from infection [41].

Iron deficiency is common in women with heavy menses due to blood loss and during pregnancy due to increased iron uptake by the developing fetus. As a result, iron supplementation is commonly prescribed to women of childbearing age and during pregnancy. An important consideration in the latter case is the increased potential for adverse maternal effects such as gestational diabetes, which have been linked to prophylactic iron supplementation [42].

The treatment and prevention of iron deficiency vary depending on the underlying cause. Dietary supplementation with iron-rich foods is an important strategy, particularly in developing countries and in individuals with restricted diets (e.g., vegans) [43]. Genetic modification of plants to enhance the bioavailability of iron may be important, particularly in areas where meat consumption is limited [44]. Iron supplements may be taken orally or in the case of ferric carboxymaltose administered intravenously [45–47]. In patients with *Helicobacter pylori* gastritis and celiac disease, treatment of the underlying condition may result in correction of anemia [25, 27–29].

### 4.4. Iron Metabolism and Laboratory Diagnosis of Iron Deficiency Anemia

Not surprisingly, the clinical features of iron deficiency and iron metabolism show considerable overlap in Figure 2, with some terms (e.g., “ferritin” and “serum ferritin”) in the clinical feature region and others (e.g., “hepcidin” and “gene”) in the iron metabolism region. The highest cited paper that addresses iron metabolism is a seminal study by Nicolas et al., which, along with subsequent work, defined and characterized the gene encoding the hepcidin protein and its regulation by anemia, hypoxia, and inflammation [48–51]. Another highly cited paper is the 2000 study by Abboud and Haile that reported the discovery characterization of the *mtp1* gene, the expression of which is upregulated in the duodenum iron deficiency and regulates intracellular iron metabolism [52].

The laboratory diagnosis of iron deficiency can generally be made using a blood specimen. Chemistry-based assays have been shown to be more sensitive than the assessment of red blood cell indices, since a substantial proportion of iron-deficient individuals do not have decreased mean corpuscular volume [1]. A battery of serum tests including ferritin (a measure of iron stores) and transferrin saturation (a measure of bioavailable iron) is most often employed. In iron deficiency, decreased serum ferritin and transferrin saturation is expected [53, 54]. The use of serum ferritin was advocated by Siimes et al. as early as 1974 [55]. Ferritin is of proven utility in the assessment of iron stores. In a study by Guyatt et al., in which serum ferritin was compared to the gold standard, iron stain of bone marrow aspirate material, a ferritin level of ≤30 *μ*g/L was of acceptable sensitivity and specificity for the detection of iron deficiency even in patients without anemia [56]. The ratio of serum transferrin receptor to serum ferritin, a potentially useful laboratory test for whole body iron stores, is limited by lack of standardization of serum transferrin receptor assays [53, 57, 58]. Other biomarkers of iron deficiency have been proposed. Serum hepcidin is potentially useful to distinguish iron deficiency anemia from anemia of chronic disease (inflammation) but has not been widely studied for this application [1]. It can be used in the diagnosis of iron-refractory iron deficiency anemia, a recessive disease due to mutation of the hepcidin inhibitor TMPRSS6, but is mainly available in referral centers [50, 59–61]. Reticulocyte hemoglobin content, which is a parameter measured by several commercially available cell analyzers, is of potential use in the diagnosis of iron deficiency anemia, although studies of its clinical applicability are currently limited [62].

### 4.5. Issues in Children

The health consequences of iron deficiency for infants and children have been well studied. In this study, the most cited paper was the first in an influential series of review articles on maternal and child malnutrition published in *Lancet* [63]. Subsequent papers in this series addressed the risk factors associated with iron deficiency and other nutritional deficiencies and potential interventions that could be employed to improve outcomes in children [64–67].

Infants born to iron-deficient mothers are at increased risk of low birth weight and prematurity [42, 68, 69]. In addition, infants and children with iron deficiency are at increased risk of defects in neural development, manifested as poor motor development, impaired cognitive development, and behavioral issues [64, 70, 71]. This is demonstrated in a study by Lozoff et al. reviewing data from Costa Rica, which found that children with hemoglobin levels ≤ 10 g/dL were particularly affected [72]. These issues are not markedly improved despite iron supplementation and can have measurable effects even up to 10 years after correction of iron deficiency [72–74]. Iron deficiency is a concern in adolescence. Halterman et al. noted decreased standardized test scores in adolescent girls with iron deficiency even in the absence of anemia [75].

One possible mechanism for the central nervous system impairment associated with iron deficiency anemia is its effects on oligodendrocytes, the principal iron-containing cells of the brain. In the presence of iron deficiency, phenomena such as impaired myelination and oxidative injury may occur [76, 77].

### 4.6. Advantages and Disadvantages of the Bibliometric Method

Our study has the following advantages. First, this is the first study using bibliometric methodology to summarize the status and development of the top 100 cited papers related to iron deficiency. Second, the top 100 cited papers related to iron deficiency in this study could be considered as benchmarks in the evolution of the field. The key papers that were retrieved by combining the reports from the WoSCC database were reviewed to ensure the accuracy and completeness of the dataset. However, like other bibliometric studies, it has limitations [4]. First, the features of the WoSCC database that allow its metadata to be used for bibliometric studies are not shared with other databases, such as PubMed, Embase, and Scopus. Thus, the studies were drawn from a single database. However, it is important to note that bibliometric analysis and keyword mapping are best approached using material from the WoSCC database [78]. Second, it would be a reasonable assumption that the citation frequency of studies published earlier should be greater than that of more recently published studies; therefore, potentially influential studies have not been considered due to their relatively low citation number. Third, other factors such as the IF of individual journals, authors, reputation, and institutions may influence whether a given study is cited.

## 5. Conclusion

This is a bibliometric study of the top 100 cited papers related to iron deficiency. The papers were published from 1964 to 2017. Based on the results from bibliometric analysis, it is clear that iron deficiency research is mainly focused on 4 areas: epidemiologic research investigating the global burden of iron deficiency, research into the clinical aspects of iron deficiency anemia, iron metabolism, and the impact of iron deficiency on children. The USA and England published the most highly cited papers in these areas. This study provides, for the first time, insights into the historical development and research status of iron deficiency using a bibliometric methodology.

## Figures and Tables

**Table 1:** Distribution of papers by country/region. Countries contributing >5 papers are shown.

Countries/regions	Records
USA	71
England	25
Switzerland	15
France	13
Italy	12
Canada	10
Australia	9
Germany	9
Greece	9
Spain	8
Japan	7
Mexico	7
Netherlands	7
Norway	7
Pakistan	7
Russia	7
Scotland	7
Sweden	7
Bangladesh	6
Brazil	6
Finland	6
India	6
Iran	6
China	6
Singapore	6

**Table 2:** Distribution of papers by organization. Organizations contributing >6 papers are shown.

Organization	Records
University of California System	30
Harvard University	15
University of London	14
University of Michigan	14
Johns Hopkins University	11
University College London	9
US Department of Veterans Affairs	9
Veterans Health Administration VHA	9
Columbia University	8
Imperial College London	8
University of Texas System	8
Case Western Reserve University	7
Emory University	7
Johns Hopkins Bloomberg School of Public Health	7
London School of Hygiene Tropical Medicine	7
Pennsylvania Commonwealth System of Higher Education PCSHE	7

**Table 3:** Journals publishing >1 of the papers in this study, impact factor, and country in which the journal is published.

Source	Titles records	Impact factor	Country
Lancet	17	60.392	UK
American Journal of Clinical Nutrition	6	6.766	USA
Blood	6	17.543	USA
Gastroenterology	5	17.373	USA
Journal of Nutrition	5	4.281	USA
New England Journal of Medicine	5	74.699	USA
Pediatrics	5	5.359	USA
Annual Review of Nutrition	3	10.897	USA
Nature	3	42.778	UK
Nature Genetics	3	27.603	UK
American Journal of Gastroenterology	2	10.171	USA
American Journal of Kidney Diseases	2	6.618	USA
Clinical Chemistry	2	7.292	USA
Gut	2	19.819	UK
JAMA Journal of the American Medical Association	2	45.540	USA

**Table 4:** Keywords occurring >9 times in papers in this study, with corresponding number of occurrences and link strength.

Term	Occurrences	Link strength
Iron deficiency	48	346
Iron	33	268
Iron deficiency anemia	31	196
Effect	28	196
Anemia	24	185
Patient	22	168
Test	19	151
Cell	18	123
Factor	18	132
Country	17	149
Diagnosis	17	117
Cause	16	153
Rate	16	175
World	15	145
Infant	14	93
Protein	14	110
Iron status	13	106
Role	13	103
Analysis	12	112
Ferritin	12	115
Number	12	125
Person	12	110
Symptom	12	102
Individual	11	110
Management	11	78
Diet	10	66
Iron deficiency anemia	10	96
Iron store	10	91
Malaria	10	90
Mechanism	10	64
Nutrition	10	69

## Data Availability

The data from this study are available from the author upon reasonable request.

## Supplementary Materials

Supplementary 1 Supplementary Data File S1: the PRISMA checklist.

Supplementary 2 Supplementary Data File S2: the data for the top 100 cited papers, organized according to number of citations and including first author name, journal, year, volume, issue, page numbers, and number of citations.
