# Supplementary material for: The Top 100 Cited Papers in the Field of Iron Deficiency in Humans: A Bibliometric Study
Source: Biomed Res Int. 2021 Jun 12;2021:5573790. doi: 10.1155/2021/5573790 (PMC8218916; doi:10.1155/2021/5573790)
Supplement: Supplementary 2 — Supplementary Data File S2: the data for the top 100 cited papers, organized according to number of citations and including first author name, journal, year, volume, issue, page numbers, and number of citations. [file 5573790.f2.docx]

**Supplemental Data File 2:** The top 100 cited papers in the field of iron deficiency in humans, organized by total number of citations

| **First author** | **Source Title** | **Design** | **Year** | **Volume** | **Issue** | **Beginning Page** | **Ending page** | **Total Citations** |
| --- | --- | --- | --- | --- | --- | --- | --- | --- |
| Vos | LANCET | Systematic analysis | 2012 | 380 | 9859 | 2163 | 2196 | 3877 |
| Vos | LANCET | Systematic analysis | 2015 | 386 | 9995 | 743 | 800 | 2791 |
| Black | LANCET | Systematic analysis/ review | 2008 | 371 | 9608 | 243 | 260 | 2705 |
| Vos | LANCET | Systematic analysis | 2016 | 388 | 10053 | 1545 | 1602 | 2220 |
| Vos | LANCET | Systematic analysis | 2017 | 390 | 10100 | 1211 | 1259 | 1351 |
| Nicolas | JOURNAL OF CLINICAL INVESTIGATION | Experimental study (mice) | 2002 | 110 | 7 | 1037 | 1044 | 1161 |
| Donovan | NATURE | Experimental study (zebrafish) | 2000 | 403 | 6771 | 776 | 781 | 1135 |
| Bhutta | LANCET | Systematic analysis | 2008 | 371 | 9610 | 417 | 440 | 1125 |
| Darmon | AMERICAN JOURNAL OF CLINICAL NUTRITION | Narrative review | 2008 | 87 | 5 | 1107 | 1117 | 1029 |
| Walker | LANCET | Narrative review | 2007 | 369 | 9556 | 145 | 157 | 978 |
| Abboud | JOURNAL OF BIOLOGICAL CHEMISTRY | Experimental study (mice) | 2000 | 275 | 26 | 19906 | 19912 | 891 |
| Anker | NEW ENGLAND JOURNAL OF MEDICINE | Prospective randomized placebo-controlled study | 2009 | 361 | 25 | 2436 | 2448 | 870 |
| McLean | PUBLIC HEALTH NUTRITION | Systematic analysis | 2009 | 12 | 4 | 444 | 454 | 869 |
| Eide | PROCEEDINGS OF THE NATIONAL ACADEMY OF SCIENCES OF THE UNITED STATES OF AMERICA | Experimental study (yeast) | 1996 | 93 | 11 | 5624 | 5628 | 858 |
| Looker | JAMA-JOURNAL OF THE AMERICAN MEDICAL ASSOCIATION | Cross-sectional study | 1997 | 277 | 12 | 973 | 976 | 856 |
| Gore | LANCET | Systematic analysis | 2011 | 377 | 9783 | 2093 | 2102 | 822 |
| Chey | AMERICAN JOURNAL OF GASTROENTEROLOGY | Guideline | 2007 | 102 | 8 | 1808 | 1825 | 771 |
| Vulpe | NATURE GENETICS | Experimental study (mice) | 1999 | 21 | 2 | 195 | 199 | 771 |
| Guranik | BLOOD | Cross-sectional study | 2004 | 104 | 8 | 2263 | 2268 | 762 |
| Ludvigsson | GUT | Guideline | 2013 | 62 | 1 | 43 | 52 | 752 |
| Robinson | NATURE | Experimental study (plant) | 1999 | 397 | 6721 | 694 | 697 | 752 |
| Rubio-Tapia | AMERICAN JOURNAL OF GASTROENTEROLOGY | Guideline | 2013 | 108 | 5 | 656 | 676 | 743 |
| James | LANCET | Systematic analysis | 2018 | 392 | 10159 | 1789 | 1858 | 742 |
| Hill | JOURNAL OF PEDIATRIC GASTROENTEROLOGY AND NUTRITION | Guideline | 2005 | 40 | 1 | 1 | 19 | 703 |
| Modell | BULLETIN OF THE WORLD HEALTH ORGANIZATION | Systematic analysis | 2008 | 86 | 6 | 480 | 487 | 699 |
| Grantham-McGregor | JOURNAL OF NUTRITION | Narrative review | 2001 | 131 | 2 | 649S | 666S | 691 |
| Lozoff | NEW ENGLAND JOURNAL OF MEDICINE | Cross-sectional study | 1991 | 325 | 10 | 687 | 694 | 676 |
| Zimmermann | LANCET | Narrative review | 2007 | 370 | 9586 | 511 | 520 | 651 |
| Pennazio | GASTROENTEROLOGY | Prospective cohort study | 2004 | 126 | 3 | 643 | 653 | 648 |
| Nicolas | PROCEEDINGS OF THE NATIONAL ACADEMY OF SCIENCES OF THE UNITED STATES OF AMERICA | Experimental study (mice) | 2002 | 99 | 7 | 4596 | 4601 | 638 |
| Yancy | CIRCULATION | Guideline | 2017 | 136 | 6 | E137 | E161 | 616 |
| Waldron | NATURE | Narrative review | 2009 | 460 | 7257 | 823 | 830 | 615 |
| Sazawal | LANCET | Prospective randomized placebo-controlled study | 2006 | 367 | 9505 | 133 | 143 | 613 |
| Lucas | CIBA FOUNDATION SYMPOSIA | Narrative review | 1991 | 156 |  | 38 | 55 | 611 |
| Walker | LANCET | Narrative review | 2011 | 378 | 9799 | 1325 | 1338 | 593 |
| Kassebaum | BLOOD | Systematic analysis | 2014 | 123 | 5 | 615 | 624 | 582 |
| Punnonen | BLOOD | Prospective cohort study | 1997 | 89 | 3 | 1052 | 1057 | 575 |
| Haas | JOURNAL OF NUTRITION | Systematic review | 2001 | 131 | 2 | 676S | 688S | 543 |
| Ganz | BIOCHIMICA ET BIOPHYSICA ACTA-MOLECULAR CELL RESEARCH | Narrative review | 2012 | 1823 | 9 | 1434 | 1443 | 540 |
| Allen | AMERICAN JOURNAL OF CLINICAL NUTRITION | Narrative review | 2000 | 71 | 5 | 1280S | 1284S | 538 |
| Lozoff | PEDIATRICS | Cross-sectional study | 2000 | 105 | 4 | E51 | - | 527 |
| Levin | AMERICAN JOURNAL OF KIDNEY DISEASES | Guideline | 2006 | 47 | 5 | S9 | S145 | 526 |
| Lozoff | NUTRITION REVIEWS | Narrative review | 2006 | 64 | 5 | S34 | S43 | 515 |
| Webster | LANCET | Narrative review | 2017 | 389 | 10075 | 1238 | 1252 | 512 |
| Engle | LANCET | Narrative review | 2007 | 369 | 9557 | 229 | 242 | 512 |
| Gloy | BMJ-BRITISH MEDICAL JOURNAL | Systematic review/ meta-analysis | 2013 | 347 |  | F5934 | - | 510 |
| Hotez | PLOS MEDICINE | Guideline | 2006 | 3 | 5 | 576 | 584 | 504 |
| Rostom | GASTROENTEROLOGY | Systematic review | 2006 | 131 | 6 | 1981 | 2002 | 491 |
| Beard | JOURNAL OF NUTRITION | Narrative review | 2001 | 131 | 2 | 568S | 579S | 491 |
| Kobayashi | ANNUAL REVIEW OF PLANT BIOLOGY | Narrative review | 2012 | 63 |  | 131 | 152 | 490 |
| Ganz | BLOOD | Validation study | 2008 | 112 | 10 | 4292 | 4297 | 476 |
| Goyer | ANNUAL REVIEW OF NUTRITION | Narrative review | 1997 | 17 |  | 37 | 50 | 472 |
| Cook | BLOOD | Method paper | 2003 | 101 | 9 | 3359 | 3364 | 467 |
| Connor | GLIA | Narrative review | 1996 | 17 | 2 | 83 | 93 | 460 |
| Plum | INTERNATIONAL JOURNAL OF ENVIRONMENTAL RESEARCH AND PUBLIC HEALTH | Narrative review | 2010 | 7 | 4 | 1342 | 1365 | 457 |
| Dewey | MATERNAL AND CHILD NUTRITION | Systematic review | 2008 | 4 |  | 24 | 85 | 442 |
| Shayeghi | CELL | Experimental study (mice) | 2005 | 122 | 5 | 789 | 801 | 441 |
| Yancy | JOURNAL OF THE AMERICAN COLLEGE OF CARDIOLOGY | Guideline | 2017 | 70 | 6 | 776 | 803 | 436 |
| Rockey | LANCET | Prospective cohort study | 2005 | 365 | 9456 | 305 | 311 | 435 |
| Finberg | NATURE GENETICS | Family study | 2008 | 40 | 5 | 569 | 571 | 422 |
| Jeney | BLOOD | Experimental study (human) | 2002 | 100 | 3 | 879 | 887 | 421 |
| Bailey | JOURNAL OF NUTRITION | Questionnaire | 2011 | 141 | 2 | 261 | 266 | 420 |
| Colangelo | PLANT CELL | Experimental study (plant) | 2004 | 16 | 12 | 3400 | 3412 | 420 |
| Scrimshaw | AMERICAN JOURNAL OF CLINICAL NUTRITION | Narrative review | 1997 | 66 | 2 | 464 | 477 | 415 |
| Georgieff | AMERICAN JOURNAL OF CLINICAL NUTRITION | Narrative review | 2007 | 85 | 2 | 614S | 620S | 413 |
| Dube | GASTROENTEROLOGY | Systematic review | 2005 | 128 | 4 | S57 | S67 | 408 |
| McLean | FOOD AND NUTRITION BULLETIN | Systematic analysis | 2008 | 29 | 2 | S38 | S51 | 407 |
| Oppenheimer | JOURNAL OF NUTRITION | Narrative review | 2001 | 131 | 2 | 616S | 633S | 404 |
| Baker | PEDIATRICS | Narrative review | 2010 | 126 | 5 | 1040 | 1050 | 398 |
| Du | SCIENCE | Experimental study (mice) | 2008 | 320 | 5879 | 1088 | 1092 | 394 |
| Abbaspour | JOURNAL OF RESEARCH IN MEDICAL SCIENCES | Narrative review | 2014 | 19 | 2 | 164 | 174 | 380 |
| Locatelli | NEPHROLOGY DIALYSIS TRANSPLANTATION | Guideline | 2004 | 19 |  | 1 | 47 | 379 |
| Bruner | LANCET | Prospective randomized placebo-controlled study | 1996 | 348 | 9033 | 992 | 996 | 376 |
| Rockey | NEW ENGLAND JOURNAL OF MEDICINE | Prospective cohort study | 1993 | 329 | 23 | 1691 | 1695 | 375 |
| Ohgami | NATURE GENETICS | Experimental study (mice) | 2005 | 37 | 11 | 1264 | 1269 | 374 |
| Bloom | WORLD DEVELOPMENT | Narrative review | 2004 | 32 | 1 | 1 | 13 | 372 |
| Beard | ANNUAL REVIEW OF NUTRITION | Narrative review | 2003 | 23 |  | 41 | 58 | 372 |
| Camaschella | NEW ENGLAND JOURNAL OF MEDICINE | Narrative review | 2015 | 372 | 19 | 1832 | 1843 | 370 |
| Graham | CLINICAL GASTROENTEROLOGY AND HEPATOLOGY | Prospective randomized placebo-controlled study | 2005 | 3 | 1 | 55 | 59 | 364 |
| Zuckerman | GASTROENTEROLOGY | Narrative review | 2000 | 118 | 1 | 201 | 221 | 361 |
| Raven | PHOTOSYNTHESIS RESEARCH | Narrative review | 1999 | 60 | 44230 | 111 | 149 | 361 |
| Ponikowski | EUROPEAN HEART JOURNAL | Prospective randomized placebo-controlled study | 2015 | 36 | 11 | 657 | 668 | 359 |
| Chey | JAMA-JOURNAL OF THE AMERICAN MEDICAL ASSOCIATION | Systematic review | 2015 | 313 | 9 | 949 | 958 | 359 |
| Forhecz | AMERICAN HEART JOURNAL | Prospective cohort study | 2009 | 158 | 4 | 659 | 666 | 356 |
| Scholl | AMERICAN JOURNAL OF CLINICAL NUTRITION | Prospective cohort study | 1992 | 55 | 5 | 985 | 988 | 352 |
| Goddard | GUT | Guideline | 2011 | 60 | 10 | 1309 | 1316 | 351 |
| Shaw | NATURE | Experimental study (zebrafish) | 2006 | 440 | 7080 | 96 | 100 | 349 |
| Allen | JOURNAL OF CLINICAL NEUROPHYSIOLOGY | Narrative review | 2001 | 18 | 2 | 128 | 147 | 333 |
| Scholl | AMERICAN JOURNAL OF CLINICAL NUTRITION | Narrative review | 2005 | 81 | 5 | 1218S | 1222S | 332 |
| Balarajan | LANCET | Narrative review | 2011 | 378 | 9809 | 2123 | 2135 | 330 |
| Okeeffe | AGE AND AGEING | Prospective randomized study | 1994 | 23 | 3 | 200 | 203 | 329 |
| Halterman | PEDIATRICS | Cross-sectional study | 2001 | 107 | 6 | 1381 | 1386 | 327 |
| Fishbane | AMERICAN JOURNAL OF KIDNEY DISEASES | Prospective randomized study | 1995 | 26 | 1 | 41 | 46 | 327 |
| Brugnara | CLINICAL CHEMISTRY | Narrative review | 2003 | 49 | 10 | 1573 | 1578 | 326 |
| Guyatt | JOURNAL OF GENERAL INTERNAL MEDICINE | Systematic review | 1992 | 7 | 2 | 145 | 153 | 325 |
| Thurnham | AMERICAN JOURNAL OF CLINICAL NUTRITION | Meta-analysis | 2010 | 92 | 3 | 546 | 555 | 323 |
| Prasad | MOLECULAR MEDICINE | Narrative review | 2008 | 14 | 44322 | 353 | 357 | 323 |
| Thomas | CLINICAL CHEMISTRY | Reference range study | 2002 | 48 | 7 | 1066 | 1076 | 313 |
| Ferguson | JOURNAL OF LABORATORY AND CLINICAL MEDICINE | Reference range study | 1992 | 119 | 4 | 385 | 390 | 312 |
| Lopez | LANCET | Narrative review | 2016 | 387 | 10021 | 907 | 916 | 308 |
